# P-427. Treatment of Mycoplasma pneumoniae infections: The Role of Non-Macrolide Antibiotics

**DOI:** 10.1093/ofid/ofaf695.643

**Published:** 2026-01-11

**Authors:** Amira Said, Ankhi Dutta, Denver Niles, Margaret Danner, Beenish Rubbab

**Affiliations:** Baylor College of Medicine, Houston, Texas; Texas Children's Hospital and Baylor College of Medicine, Houston, TX; Baylor College of Medicine, Houston, Texas; Baylor College of Medicine, Houston, Texas; Baylor College of Medicine, Houston, Texas

## Abstract

**Background:**

*Mycoplasma pneumoniae* is an important cause of pneumonia and extrapulmonary disease in children and adolescents. In the past 2 years, we have seen an increase in the prevalence of *M. pneumoniae* infections in children. Macrolide antibiotics are the first line of therapy. The resistance rate of *M. pneumoniae* ranges from 1% to 21% in the United States whereas in Asia, it is as high as 90% in some regions. We aimed to evaluate indications for the use of non-macrolide antibiotics in the treatment of symptomatic *M. pneumoniae* infections at our institution.

**Figure:**
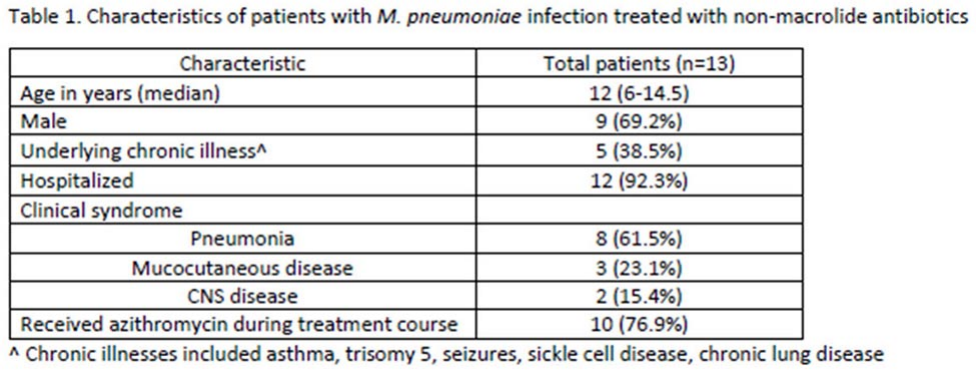

**Methods:**

Retrospective chart review of the electronic medical record was completed on all symptomatic patients in our hospital system who had a positive nasal or nasopharyngeal polymerase chain reaction (PCR) for *M. pneumoniae* from October 2023 to January 2025. This included patients with community acquired pneumonia and extrapulmonary manifestations such as mucocutaneous disease and neurologic disease. Data was collected on demographics, clinical course, treatment and outcomes including reasons for receiving an antibiotic other than azithromycin such as severity of illness, presence of CNS disease, lack of response, cost and allergy.

**Results:**

Among 84 patients with confirmed *M. pneumoniae* by PCR, 75% were hospitalized and 35.7% required supplemental oxygen. Fever (86.9%) and cough (82.1%) were the most common presenting symptoms. Seventy-six patients (90.5%) were started on azithromycin and 13 patients (15.7%) were treated with an antimicrobial in place of or in addition to azithromycin (Table 1). Eight patients were treated with doxycycline and three were treated with levofloxacin. The median duration of non-macrolide antibiotic use was 7 days (IQR 6-10.5 days). Non-macrolide use was associated with disease severity or lack of azithromycin response.

**Conclusion:**

Despite a significant proportion of patients requiring hospitalization, most patients received a macrolide for treatment of *M. pneumoniae* infection with good outcomes. Use of non-macrolide agents was limited and driven by clinical severity or lack of improvement.

**Disclosures:**

Denver Niles, MD, BioMerieux: Advisor/Consultant

